# The First Subscribers to the First Dental Journal

**Published:** 1898-05

**Authors:** 


### The First Subscribers to the First Dental Journal.

At a recent meeting of the Odontographic Society, of Chicago, in a paper read before that body, some reference was made to the origin of journalism in the dental profession.

The *American Journal of Dental Science* was the first dental journal published in the world. Some reference was made in the discussion of the subject to the interest manifested by the members of the profession in this the first journalistic enterprise. Reference was made to the number of subscribers and the warm interest manifested by many of them in support of the journal. The journal was published quarterly at $5.00 per year. It will be seen by this list that many subscribed for more than one volume each, some taking forty copies, others twenty, ten, etc.

It was furthermore suggested that the names of these supporters of the first journal be placed upon permanent record, in as much as this list is now accessible to but very few in the profession.

Not one in five hundred of the practicing dentists of the country, perhaps, would know now where to find the names of these persons.

Dr. G. V. Black was the first to suggest the publication of these names, and doubtless many will look over them with more than an ordinary interest.

Ayers, Daniel, Amsterdam, Mont. County, N. Y., 1
Allen, Richard L., Saratoga Springs, 1
Alcock, James, N. Y., 2
Austin, George, Baltimore 1
Austin, Nathan'l, Harrisonburg, Va., 2
Ash, Claudius, London, 2
Azling, Isaac, M. D., Boston, 1
Auten, F. P., Lambertville, N. J., 1
Atkinson, John, Leeds, Eng. 1
Arthur, Robert, Baltimore, 1
Andrews, E. H., Charlotte C. H., N. Carolina, 1
Ash, G. E., London, 1
Arnold, Wm., M.D., N. Y. 1
Avery, Samuel, N. Orleans, 1
Brewster, C. S., Paris, France 1
Burdell, John, N. Y., 20
Bridges, M. K., Brooklyn, 20
Baker, Elisha, N. Y., 20
Brown, A. W., N. Y., 20
Bryan, Elijah, N. Y., 1
Blake, Elihu, N. Y., 1

Bancroft, T. L., Granville, Ohio, 1
Brown, B. B., St. Louis, Mo. 2
Backus, G., Nashville, Tenn. 1
Briscoe, A. W., Baltimore, 1
Barstow, Wm. H., M. D., Georgetown, Ky., 1
Buchan, David L., Hempstead, Md., 1
Bidgood, Rich'd W., Smith, Va., 1
Badger, Felix H., Decatur Ga., 1
Brown, Solyman, N. Y., 5
Becht, A. J., Hague, Netherlands, 1
Bareud, J. Dent., George St., Manchester, 1
Barend, Sam'l, Liverpool, Eng., 1
Bradford, D., Augusta, Ky., 1
Bell, Thomas, London, 1
Ballanger, D. W., Montgomery, Ala., 2
Brockway, Josephus, Troy, 2
Blanding, S., M. D., Columbia, S. C., 1
Baldwin, James Oscar, Newark, N. J., 1
Brown, Chas. D., Philadelphia, 1
Burdell, Harvey, M. D., N. York, 2
Bliss, S., M. D., Syracuse, N. York, 1
Buck, J. B., N. York 1
Briscoe, Jas. H., Phila. 1
Boyken, F. M., Smithfield, Va. 1
Burr, Hudson, Philadelphia 1
Ballard, Geo. W., Madison, Morgan Co., Ga. 1
Bull, Abel, M. D., Boston 1
Bemis, S. A., Boston 1
Budd, John D., Mount Holly, N. J. 1
Burr, Wm. H., Mount Holly, N. J. 1
Cook, Doctor, Brooklyn 1
Clute, Nicholas, Louisville, Ky. 2
Chandler, James, Schenectady, N. Y. 1
Cobb, B. C., M. D., Clarkstore, Martins Co., N.C. 1
Cox, A. L., M. D., New York 1
Cleveland, J. A., Charleston, S. C. 1
Crocker, Fred'k, Sag Harbour, L. I. 1
Clark, F. H., Baltimore 1
Comegys, —— Baltimore 1
Cutler, Wm. Daily, Baltimore 1
Cutler, Sam'l Jackson, Baltimore 1
Coleman, R. K., Baltimore 1
Cauling & Edmunds, Baltimore 1
Cobb, Anson, Brooklyn 1
Clark, C., Jacksonville, Ill. 1
Clark, James, Springfield, Ill. 1

Cassill, J. F., Upper Marion Col. Mo. 1 1
Chewning, F. B., Rich., Va. 2
Clark, James, Lebanon, O. 1
Coppel, Chas., Preston, Eng. 1
Crane, O. P., Geneva, N. Y. 1
Chevalier, J. D., N. Y. 1
Crofoot, E. E., Middletown, Conn. 1
Crofoot, L. L., Middletown, Conn. 1
Candee, J. G., Troy, N. Y. 1
Clark, A., Penyan, N. Y. 1
Cameron, James, Philadelphia 1
Copeland, W. S., M. D., Rich Square, Virginia 1
Carter, J. H., Ravenna, Portage Co., O. 1
Caldwell, Geo. H., Rushville, Ia. 1
Camp, W. C., Oxford, Granville Co., N. Carolina 1
Cuyler, Vernon, M. D., Hartford, Conn. 1
Caldwell, D., Philadelphia 1
Copelin, J., New York 1
Crane, W. S., Hartford, Conn. 1
Culp, Clark, Philadelphia 1
Dexter, Doctor, Brooklyn 1
Dunning, H. H., Buffalo 1
Davis, W. H. H., Cassville, Oneida Co., N.Y. 1
Duncan, Archibald, N. Y. 1
Davesson, Frederick A., M. D., Hillsboro, London Co., Va. 1
Dunlap, Joseph, Chillicothe, O. 1
Dunbar, J. R. H., M. D., Balt. 1
Desha, John R., M. D., Little Rock, Arkansas 1
De Loude, Le Chas., Wolverhampton, England 1
Dodge, J. Smith, N. Y. 1
Dewar, Henry, Edinburgh, Scotland 2
Dodge, Andrew, Matanzas, Cuba 1
Dixon, Rufus E., M. D., Boston 1
Doolittle, A. B., Plymouth, Conn. 1
Esterly, D., M. D., Troy, New York 1
Early, —— Doctor, Lynchburg, Va. 2
Elmendorf, Joseph, Pennyan, N. Y. 1
Ellery, E., Baltimore 1
Evans, J. N., M. D., Cynthiana, Ky. 1
Elliott, Joseph D., Leicester, Mass. 1
Ensor, —— Dentist, Liverpool 1
Ellis, Calvin, M. D., Boston 1
Evans, G. W., Cincinnati 1
Epps, W. J., M. D., Langhorne's P. O., Virginia 1
Evans, Thos. W., Philadelphia 1
Easton, Wm. T., Providence, R. I. 1

Fenn, Horatio N., M. D., Rochester 2
Foster, J. H., M. D., New York 2
Flagg, Josiah F. M., M. D. Boston 1
Frazer, Doctor, Cynthiana, Harrison Co., Ky. 1
Follen, John H., North. C. H., Va. 1
Frink, J. N., Portland, N. H. 1
Foote, George, Vernon, N.Y. 1
Faulkner & Pierpont, Man. England 1
Fundenberg, G. B., Dentist Pittsburgh, Pa. 1
Franklin, B. W., Fairfield, Herkimer Co., N. Y. 1
Fraetas, J. A., New York 20
Fay, Timothy, Baton Rouge, La. 1
Fay, Solomon, Chester Factories, Mass. 1
Ferguson, Jas. H., Northumberland C. H., Va. 1
Fouche, W. W., Philadel- 1 phia 1
Falconer, John, New York 1
Gilfiland, Doctor, Brooklyn 1
Gaines, Rich. W., Charlotte C. H., Virginia 1
Garrison, Doctor, Brooklyn 1
Gallop, L. F., Newport, R. I. 1
Gardette, E. B., Philadelphia 2
Green, L. T., Nashville, Tenn 1
Goddard, W. H., Louisville, Ky. 1
Gill, Bryson, Baltimore 1
Gunnell, Jas. S., M. D., Washington 1
Griffith, S. & E., Louisville, Ky. 1
Geer, Rev. John A., Maryland 1
Grandhomme, M. P., Paris, France 1
Gidney, Eleazar, Man., Eng. 1
Greenwood, Isaac J., New York 40
Grant, C. W., Newburg, New York 1
Greenleaf, Chas., Hartford, Conn. 1
Ganson, Holton, Batavia, New York 1
Gaines, B. B., Cassville, Ga. 1
Githens, John H., Phila. 1
Gunn, —— Nashville, Tenn. 1
Grimes, Doctor, Greenborough, Georgia 1
Herd, Doctor, Brooklyn 1
Harris, Chapin A., M. D., Baltimore 40
Hawes, Arnold, Pawtucket, R. I. 1
Hulihen, J. P., Wheeling, W. Va. 1
Hawes & Allen, New York 1
Houston, P., New York and Charleston 1
Hartness, Thos. L., New York 1

Hewlet, J. W., Greensboro, North Carolina 1
Holmes, Oliver, Baltimore 1
Howard, F., Washington City 1
Harris, John, M.D., Georgetown, Ky. 2
Hall, A. S., M.D., Scotland Neck, North Carolina 1
Hubberd, E. R., Newbern, North Carolina 1
Harper, Sam'l, Kent Island, Maryland 1
Hand, G. C., Easton, Pa. 1
Humphreys, Geo. W., Winchester, Virginia 1
Hodgson, Doctor, Whiteplains, New York 1
Harrison, R. H., M. D., Huntsville, Alabama 1
Helsby, ——, Manchester, England 1
Hudson, Edward, Philadelphia 1
Hamlin, T. B., Wythville, Virginia 1
Hallified, Doctor, Petersburg, Virginia 1
Hought, Chas. J., Philadelphia 1
Hughes, H. W., Westminster, Maryland 1
Hill, A., Norwalk, Conn. 1
Hoit, Emmett M., Stanwich, Connecticut 1
Halleyman, W. F., Maytinton, S. Carolina 1
Imrie, ——, Dentist, Manchester, England 1
Ingraham, Thomas, Phila. 1
Johnson, Wm., Hagerstown, Maryland 2
Jenks, W. D., Fredericks-town. Maryland 2
J'ollen, John N., York C. H., Virginia 1
King, Doctor, Brooklyn 1
Kelley, Elbridge G., Newburyport, Massachusetts 2
Knower, Daniel, New York 1
Keen, Benjamin F., Hills, Ga. 1
Knapp, F. H., Baltimore 1
Kearsing, George, New York 20
Kingsbury, C. A., Philadelphia 1
Kimball, Horace, New York 1
Keemy, B. M., Hudson, N.Y. 1
Koecker, Leonard, M. D., London 2
Keene, Doct., Newtown, Scott Co., Kentucky 1
Latham, Hiram, Brooklyn 1
Lum, John, Patterson, N. J. 1
Levett, M., New York 1
Lovejoy, John, New York 1
Laroque, Edward, Baltimore 1
Lapham, B. B., Baltimore 1
Lawrence, J., M. D., Tarboro, N. C. 1
Leadbetter, John, Alexan., D. C. 1
Lyon, S. K., N. Orleans 4
Llloyd, T. B., Manchester, England 1

Lloyd, Rich., Liv. Eng. 1
Laird, O. P., Columbus, Ga. 1
Lamphire, Wm., Alexan., D. C. 1
Lawrence, S. W., Philadelphia 1
Lee, Joseph, M.D., Camden, S. C. 1
Lawrence, E. B., Crawfordsville, Ga. 1
Loomis, J. C., Carlisle, Pa. 1
Latimer, James, Madison, Morgan Co., Georgia 1
Lethbridge, Sam'l, Richmond, Va. 1
Marvin, Doctor, Brooklyn 1
Miller, Seth P., Worcester, Mass. 1
Maynard, Ewd., M. D., Washington City 20
Martin, Chas. F., Norfolk, Va. 1
M'Kinney, W. N., Fredricksburg, Va. 20
Milhau, John, New York 1
Munson, W. G., New Haven 1
Manly, Horace, Canada, N. Y. 1
Macall, Leonard, M. D., Baltimore 1
Miller, J. H., Professor, etc., Baltimore 1
Merryman, Geo., Baltimore 1
Manning, M. E., Tarboro, North Carolina 2
M'Cabe, James D., Richmond, Va. 2
McDonald, G., M.D., Macon, Georgia 2
Macey, Wm. M., M. D., White Sulphur, Scott Co., Kentucky 1
McAllister, J. M., Albany 1
McNaughton, M. A., Albany 1
Mason, Doctor, Brooklyn 1
Merritt, C., Bridgeport, Con. 1
Martin, J., Portsmouth, Eng. 1
MacPherson, James, Glasgow, Scot. 1
Middleton, Ellis, Philadelphia 1
McIlhinney, Joseph E., Philadelphia 1
Matson, Alpheus, Auburn, New York 1
M'Grath, R., Philadelphia 1
More, Justus E., Philadelphia 1
Middleton, Wm., New York 1
Merritt, Chas., Bridgeport, Conn. 1
Murrill, L., Petersburg, Va. 1
Nelson, Alexander, Albany 1
Norman, S. P., Little Rock, Arkansas 4
Neal, D., Philadelphia 1
Noyes, Enoch, Baltimore 20
Nasmyth, Robert, Edinburgh, Scotland 1
Overfield, M., Winchester, Virginia 2
Parker, Doctor, Brooklyn 1
Parmly, L. S., N. Orleans 20
Parmly, Jahial, New York 20

Parmly, Jahial, Savannah 1
Parmly, Geo. W., N. Orleans 1
Parmly, David, New York 1
Parmly, Eleazar, New York 40
Parmly, Rudolph, Mobile 1
Parmly, W. Sam'l, N. York 1
Patello, Wm. H., Charlotte C. H., Virginia 1
Park, David N., A. M., New York 1
Parkhurst, Wm. H., N. Y. 1
Peak, J. M., Cooperstown, New York 1
Plant, Ebenezer, Willimantic, Conn. 1
Pritchard, P. C., Jackson, North Carolina 1
Plough, A. L., New Orleans 1
Parsons, J. H., Liverpool, England 1
Palmer, W. A., Stratford, Connecticut 1
Pleasants, Chas., Plainesville, O. 1
Perkins, Jacob, Springfield, Mass. 1
Parker, T. H., Philadelphia 1
Pent, George, M.D., Lawrenceville, Virginia 1
Pancost, S., Shelbyville, Ky. 1
Royce, W. A., Poughkeepsie, New York 3
Robinson, E. C., Norfolk, Va. 1
Roper, Lewis, M. D., Philadelphia 20
Rowell, Chas., New York 2
Roe, Early, M. D., Hillsborough, Ga. 1
Rodriguez, B. A., M. D., Charles, S. C. 1
Ritter, ——, Washington City 1
Robinson, E., M. D., Leesburg, Ky 1
Reynolds, Wm., M. D., Camden, S. C. 1
Reinstein, Frederick, Philadelphia 1
Ross, Samuel, New York 1
Root, J. B., Hamilton, Madison Co., New York 1
Reese, F. A., M. D., Hamilton, Bermuda 1
Strickland, Benj., Cleveland, O. 1
Snow, R. J., Buffalo 1
Smith & Thackston, Farmville, Va. 1
Scott, W. R., Raleigh, N. C. 4
Shuff, P. L., M. D., Leesburg, Ky. 1
Sneeds, M. D., Frankfort, Kentucky 1
Stringfellow, S. L., Balt. 20
Stratton, Chas., Keene, N.H. 1
Sanders, M., London 1
Sanborn, E., Andover, Mass. 1
Smith, Wm., Liverpool, Eng. 1
Stinkley, B. D., Albany 1
Smith, G. W., New Orleans 1
Stuart, R., Knoxville, E. T. 1
Smith, J. W., Amherst, Mass. 1
Searle, F., Springfield, Mass. 1
Stockton, S. W., Philadelphia 20
Stowell, John, Philadelphia 1

Shepherd, S. M., Petersburg, Va. 1
Sherman, A., Newark, N. J. 2
Simpson, ——, Manchester, England 1
Sale, T. A., Williamsboro, North Carolina 1
Sims, J. M., M. D., Fish Dam, Union Dis., S. C. 1
Sanders, E., 16 Argyle st. London 1
Stevens, B. H., Elbridge, Onondaga Co., N. Y. 1
Taylor, Edward, M. D., Bainbridge, O. 2
Tucker, E. G., M. D., New York 2
Trenor, John, M. D., New York 1
Trenor, James, M. D., N. Y. 1
Tilyard, H. W., Baltimore 1
Taliaferro, T. S., M. D., Maysville, Ky. 1
Thompson, ——, Doctor, Columbus, O. 1
Thorn, Doctor, Brooklyn 1
Tyler, Nathaniel, Chicapee Falls, Mass. 1
Taylor, James, Crawfordville, Ia. 1
Teter, G. T., Dentist, Greenfield, Highland Co., Ohio 1
Thorn, Doctor, Edgefield, C. H., South Carolina 1
Thackston, Doctor, Farmville, Va. 1
Townsend, Sam'l, Baltimore 1
Truman, Geo., Philadelphia 1
Van Praag, A. S., New York 1
Vincent, Ezra, New York 1
Van Camp, Louisville, Ky 1
Van Paten, C. H., Pittsburg 1
Vanboskirk, L. E., St. John, N. B. 1
Willard, M. T., Concord, New Hampshire 1
Weed, J. Sacket, W. Greenfield, N. Y. 1
White, Geo. H., New York 1
Walker & Jones, New York 20
Wanzer, N. C., Auburn, New York 1
Wayt, John G., Richmond, Virginia 2
Wilson, J. D., Richmond, Virginia 2
Ware, W., M. D., Wilmington, N. C. 1
Wheat, J. B., New Haven, 1
White, S. D., Philadelphia 1
Worthington, R. C., M. D., Murfreesboro, N. C. 1
Ward, David G., Wanesboro, N. C. 1
Ward, W. A., Petersburg, Va. 2
Willmore, E., Baltimore 1
Wheeler, E. D., Hillsboro, Coffee Co., Tenn. 1
Williams, E. C., M. D., Philadelphia 1
Wells, H., Hartford, Conn. 1
Young, H., Troy, N. Y. 1
Yard, George, Philadelphia 1

